# Supplementary material for: CXCL17 binds efficaciously to glycosaminoglycans with the potential to modulate chemokine signaling
Source: Front Immunol. 2023 Oct 24;14:1254697. doi: 10.3389/fimmu.2023.1254697 (PMC10628517; doi:10.3389/fimmu.2023.1254697)
Supplement: Supplementary file 1 [file DataSheet_1.pdf]

Supplementary Material

CXCL17 binds efficaciously to glycosaminoglycans with the potential to modulate chemokine signalling

Sean P. Giblin[1], Sashini Ranawana[1], Shyreen Hassibi[1], Holly L. Birchenough[2], Kyle T. Mincham[1], Robert J. Snelgrove[1], Tomoko Tsuchiya[4], Shiro Kanegasaki[5], Douglas Dyer[2,3] and James E. Pease[1*]

[1]National Heart and Lung Institute, Imperial College London, London, United Kingdom.

[2]Wellcome Centre for Cell-Matrix Research, Lydia Becker Institute of Immunology and Inflammation, Faculty of Biology, Medicine and Health, Manchester Academic Health Science Centre, University of Manchester, United Kingdom

[3]Geoffrey Jefferson Brain Research Centre, Manchester Academic Health Science Centre, Northern Care Alliance NHS Group, University of Manchester, Manchester, UK

[4]Research Institute, National Center for Global Health and Medicine, Shinjuku-ku, Tokyo 162-8655, Japan.

[5]Gunma University, Heavy Ion Medical Center, Maebashi City 371-8511, Japan

*** Correspondence:**

Corresponding Author j.pease@imperial.ac.uk

Supplementary Figures

Supplementary Figure 1. Increasing the rate of AlphaFold2 structural model recycling maintains the prediction that CXCL17 (24-119) has a C-terminal alpha-helix. The structure of CXCL17 was modelled with ColabFold: AlphaFold2 using various iterances of model recycling, as this has previously been described to improve the quality of structures for which low numbers of homologs are present in reference libraries. (**A**) Multiple sequence alignment (MSA) was performed against CXCL17 (24-119) with MMSeqs2 Uniref90 and environmental databases, with the sequence coverage per amino acid position of CXCL17 displayed. The structure of CXCL17 was modelled 3 times, either with 3 (**B-D**), 12 (**E-G**) or 48 (**H-J**) iterances of model recycling. Stereochemical plausibility and confidence in the models are reflected in the plDDT scores per residue position for the top 5 ranking models (**B, E, H**). Plots of predicted alignment error (PAE) per residue position for the top 5 ranking models are displayed (**C, F, I**). For each predictions with 3 and 12 iterations of model recycling, the rank 1 structure model is rotated 90˚ around the Y-axis (**D, G**). The top 5 highest ranking structural models of CXCL17 (24-119) generated with 48 iterances of model recycling are displayed (**J**). Secondary structure is indicated (α-helix; cyan, random coil; salmon pink), and all cysteines are coloured yellow.

Supplementary Figure 2. Structural prediction of CXCL17 (24-119) by C-I-TASSER questions chemokine-like homology. Structural predictions of CXCL17 (24-119) were generated by C-I-TASSER, integrating the I-TASSER hierarchical structure modelling approach with deep learning based contact predictions to guide Replica Exchange Monte Carlo (REMC) folding simulations. (**A**) Secondary structure predictions by amino acid position are presented where 'C' denotes random coil, and 'H' denotes α-helix. The top 5 ranking models by cluster size (Models 1-5); rank 1 (**B**) and ranks 2-5 (**C-F**), were rendered in pyMOL with secondary structure indicated (α-helix; cyan, random coil; salmon pink) and all cysteines coloured yellow. Confidence (C-scores) for each model are indicated. For **B**, all positively charged arginine and lysine residues were coloured blue, the model is rotated 180˚ and displayed without and with molecular surface. NH_2- and COOH- termini are annotated as N and C respectively.

Supplementary Figure 3. Structural modelling of CXCL17 (24-119) with RoseTTAFold ColabFold using MMSeqs2 reference library or single sequence input for *de novo* folding. (**A**) ColabFold RoseTTAfold structural model of CXCL17 (24-119) using MMSeqs2 and Uniref library (pdb70 template input). The MSA displays the sequence coverage of the input against homologs in the UniRef90 and environmental libraries. (**B**) Shows plDDT scores per residue, indicating local confidence in the model, and (**C**) shows the top ranking structural prediction generated in PyMOL. (**D**) ColabFold RoseTTAfold structural model of CXCL17 (24-119) using single sequence input settings for *de novo* folding of proteins with few homologs in the UniRef90 and environmental reference

libraries. plDDT scores per residue are indicated, and (**E**) shows the top ranking structural prediction generated in PyMOL. All structures are shown with rotation 90˚ on the Y-axis, with all cysteines coloured yellow, all arginines and lysines coloured blue, and secondary structure elements coloured teal (α-helix) and salmon pink (random coil). NH_2- and COOH- termini are annotated as N and C respectively.

Supplementary Figure 4. Structural modelling of CXCL17 (24-119) with AlphaFold ColabFold using single sequence input for de novo folding of proteins with low numbers of homologs in the reference library. ColabFold AlphaFold2 structural model of CXCL17 (24-119) using the single input sequence setting for de novo folding of proteins with low numbers of homologs in the UniRef90 and Environmental reference libraries. No MSA was performed, (**A**) plDDT and (**B**) PAE scores per residue for the top 5 ranking models (rank1-5) are displayed. (**C**) The top ranking structural model was generated in PyMOL and rotated 90˚ on the Y-axis. All cysteines are coloured yellow, all arginines and lysines coloured blue, and secondary structure elements coloured teal (α-helix) and salmon pink (random coil). NH_2- and COOH- termini are annotated as N and C respectively.

Supplementary Figure 5. AlphaFold2 ColabFold structural prediction of CXCL17 (24-119) homodimer displaying the top 5 ranking models. Structural predictions of CXCL17 (24-119) homodimer were generated by AlphaFold2 ColabFold using 12 iterances of model recycling. (**A**) The sequence coverage from the initial MSA step is displayed and (**B**) stereochemical plausibility and confidence in the models are reflected in the plDDT scores per residue position for the top 5 ranking models. (**C**) Plots of predicted alignment error (PAE) per residue position for the top 5 ranking models are displayed. (**D-H**) Corresponding structural predictions for models ranked 1-5 respectively. Predicted structures are displayed with all cysteines coloured yellow and secondary structure elements coloured teal (α-helix), magenta (β-sheet) and salmon pink (random coil). NH_2- and COOH- termini are annotated as N and C respectively.

Supplementary Figure 6. Production of recombinant In-house CXCL17 (24-119) . (A) 6xHis-SUMO3-CXCL17 (24-119) was purified from the soluble fraction of Shuffle T7 Express *E.coli* by IMAC with the resulting HPLC chromatogram displaying A_{280nm} absorbance and (B) SDS-PAGE with Coomassie stain displaying the distribution of total protein eluted in various fractions of the

purification. The 5 right-most lanes of the SDS-PAGE gel image show the distribution of protein at various stages of the digestion procedure of 6xHis-SUMO3-CXCL17 (24-119) with *Ulp1*. (**C**) Solubilized and refolded CXCL17 (24-119) was purified by HiTrap heparin HP 1ml affinity chromatography and (**D**) total protein eluted from the column was visualized by SDS-PAGE with Coomassie stain. Recombinant in-house generated proteins were resolved by Western blot with CXCL17 anti-serum (sheep anti-human pAb (AF4207). (**E**) serial dilutions of SUMO3-CXCL17 (24-119), (**F**) and CXCL17 (24-119) . Dimer complexes were covalently crosslinked by addition of 5 mM BS^3 chemical crosslinker to various concentrations of (G) SUMO3-CXCL17 (24-119), (H) and CXCL17 (24-119). Images are representative of n=3 separate experiments. Protein identity was also confirmed by probing with mouse anti-hCXCL17 mAb (MAB4207) (data not shown).

Supplementary Figure 7. The SUMO3 tag has modest effects on the ability of CXCL17 to bind to Heparin, HS or CS. The N-terminal SUMO3-tag on SUMO3-CXCL17 (24-119) and the C-terminally truncated mutants was confirmed not to interact with binding of CXCL17 (24-119) to GAGs using a colorimetric static-binding assay. Preparations of CXCL17 (24-119) , SUMO-CXCL17 (24-119) and SUMO3-tag were immobilised, to which the binding of 1µg/ml biotinylated GAGs heparin (**A**), HS (**B**) or CS (**C**) were quantified (n=4). Data were normalized to maximum binding recorded per experiment and display mean ±SEM. (**D-F**) 500 nM CXCL17 (24-119) and SUMO-CXCL17 (24-119) were immobilised in both their native state and after being reduced with 2 mM DTT, to which the binding of 1µg/ml heparin, HS or CS was quantified (n=3). Data displayed as mean +SEM. Two-way ANOVA with multiple comparisons and Dunnett's post-test was performed and no statistical significance was detected (NS).

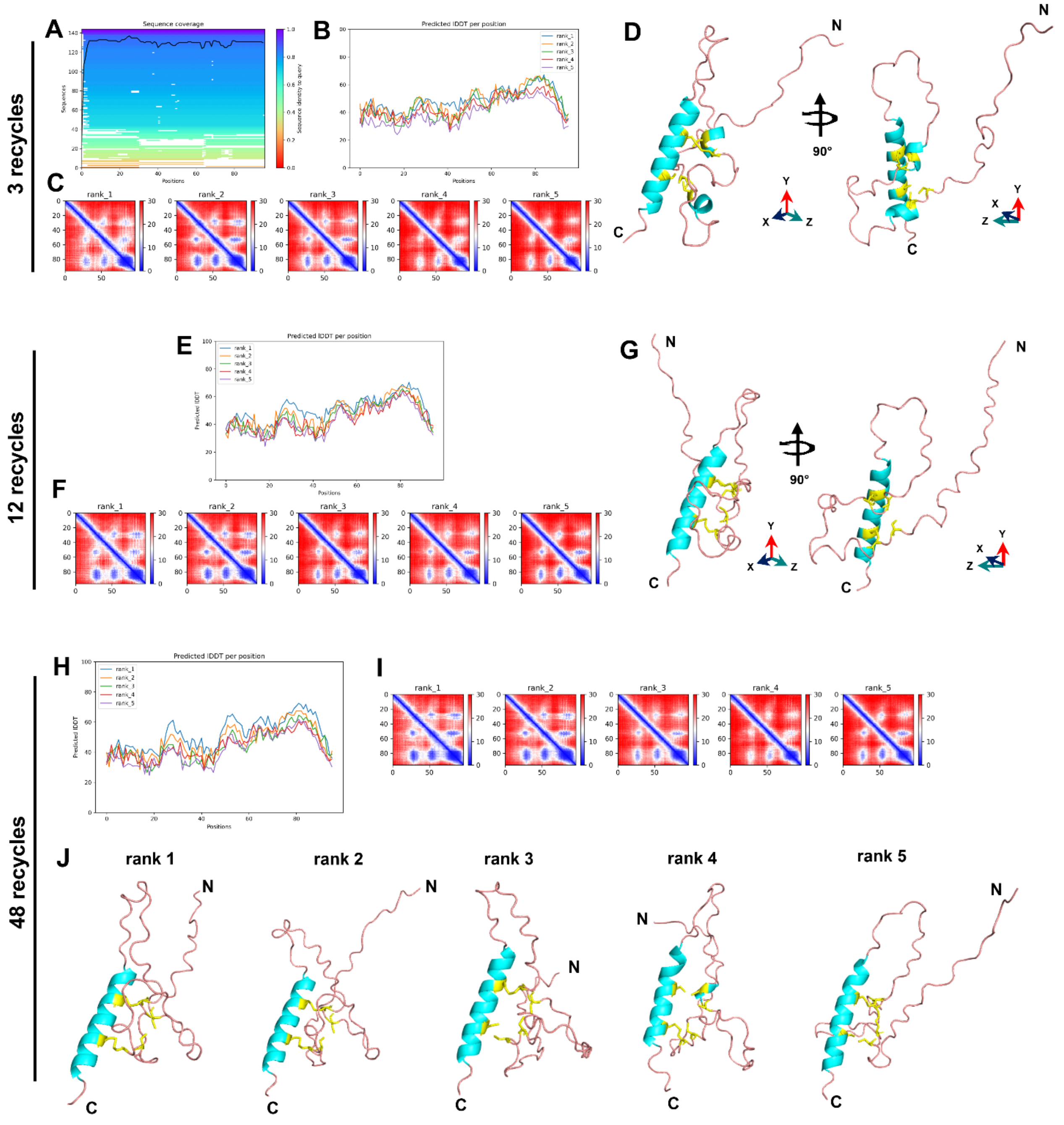

Supplementary Figure 1.

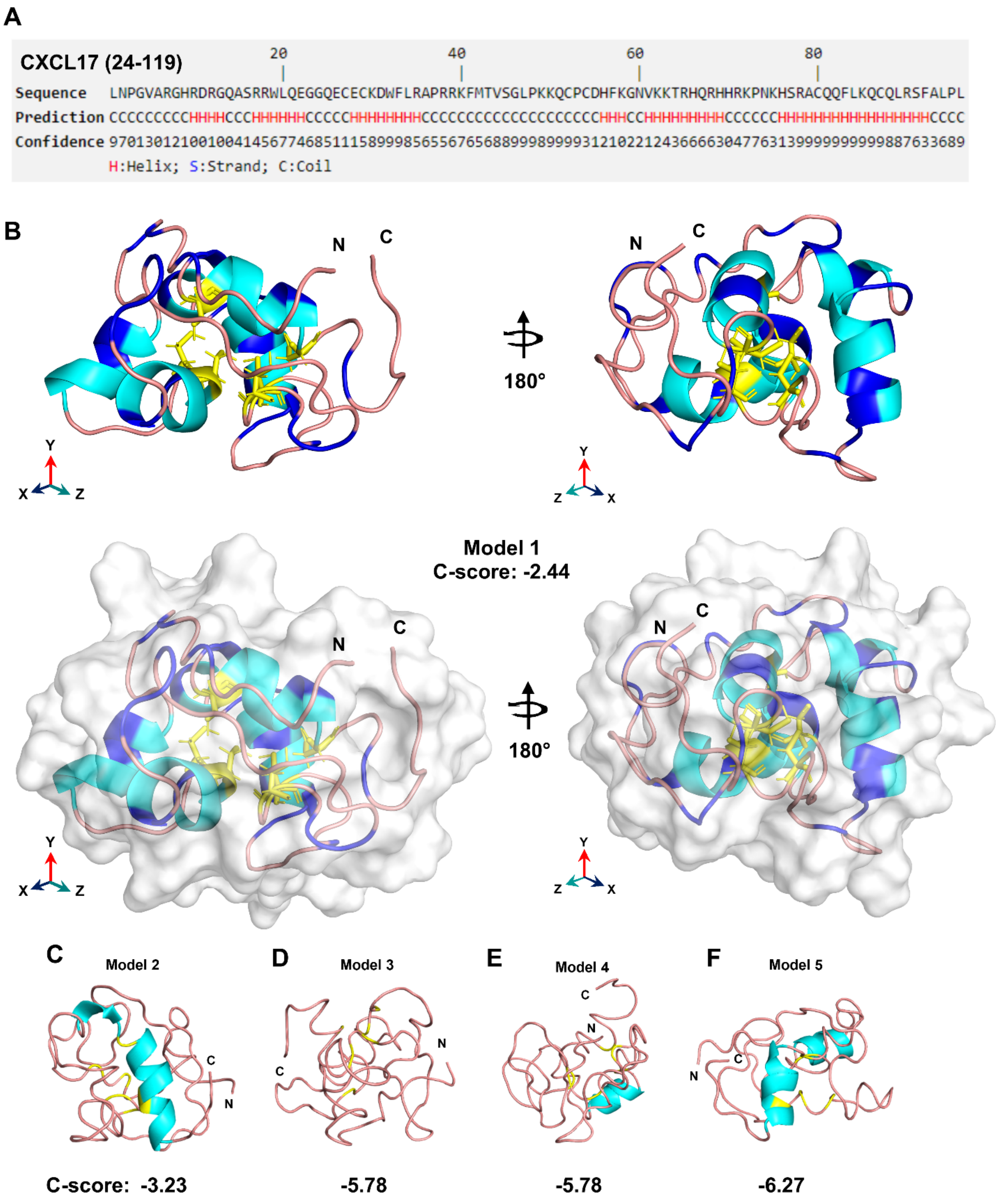

Supplementary Figure 2.

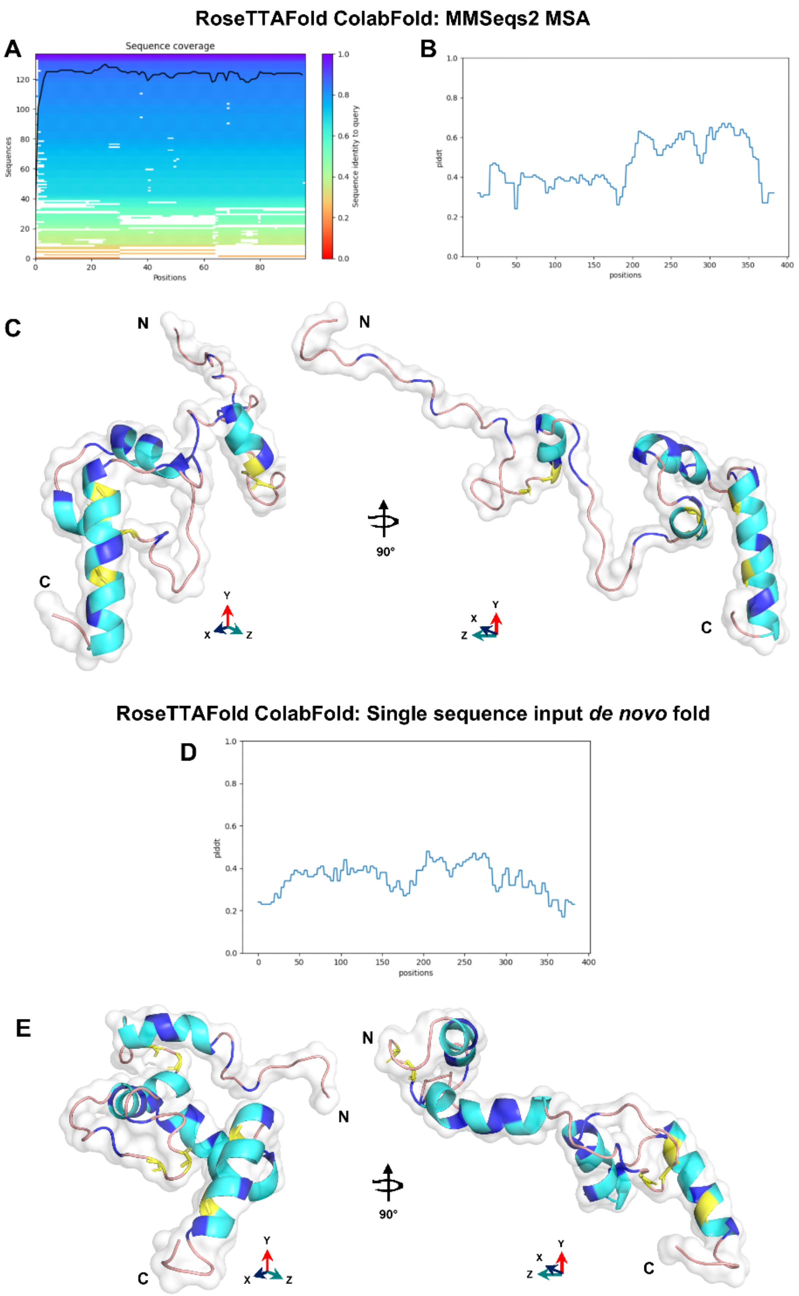

Supplementary Figure 3.

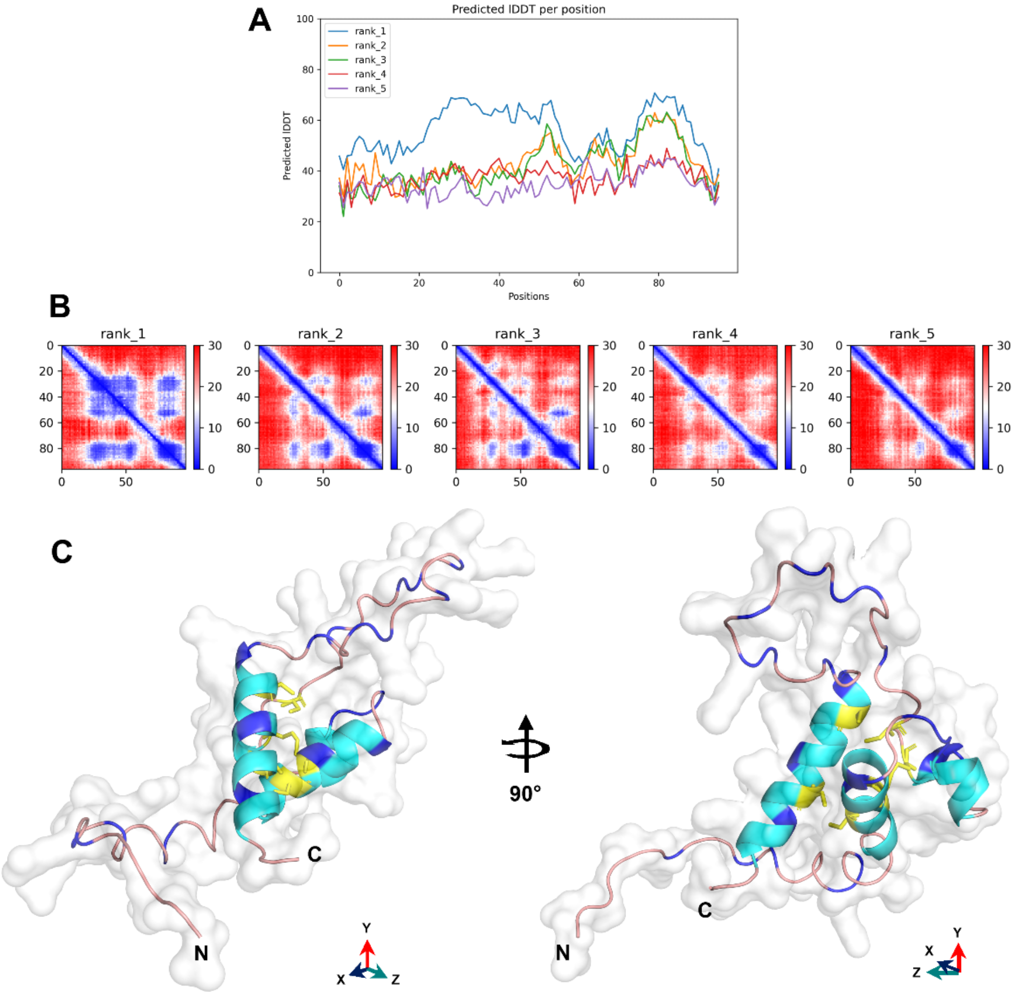

Supplementary Figure 4.

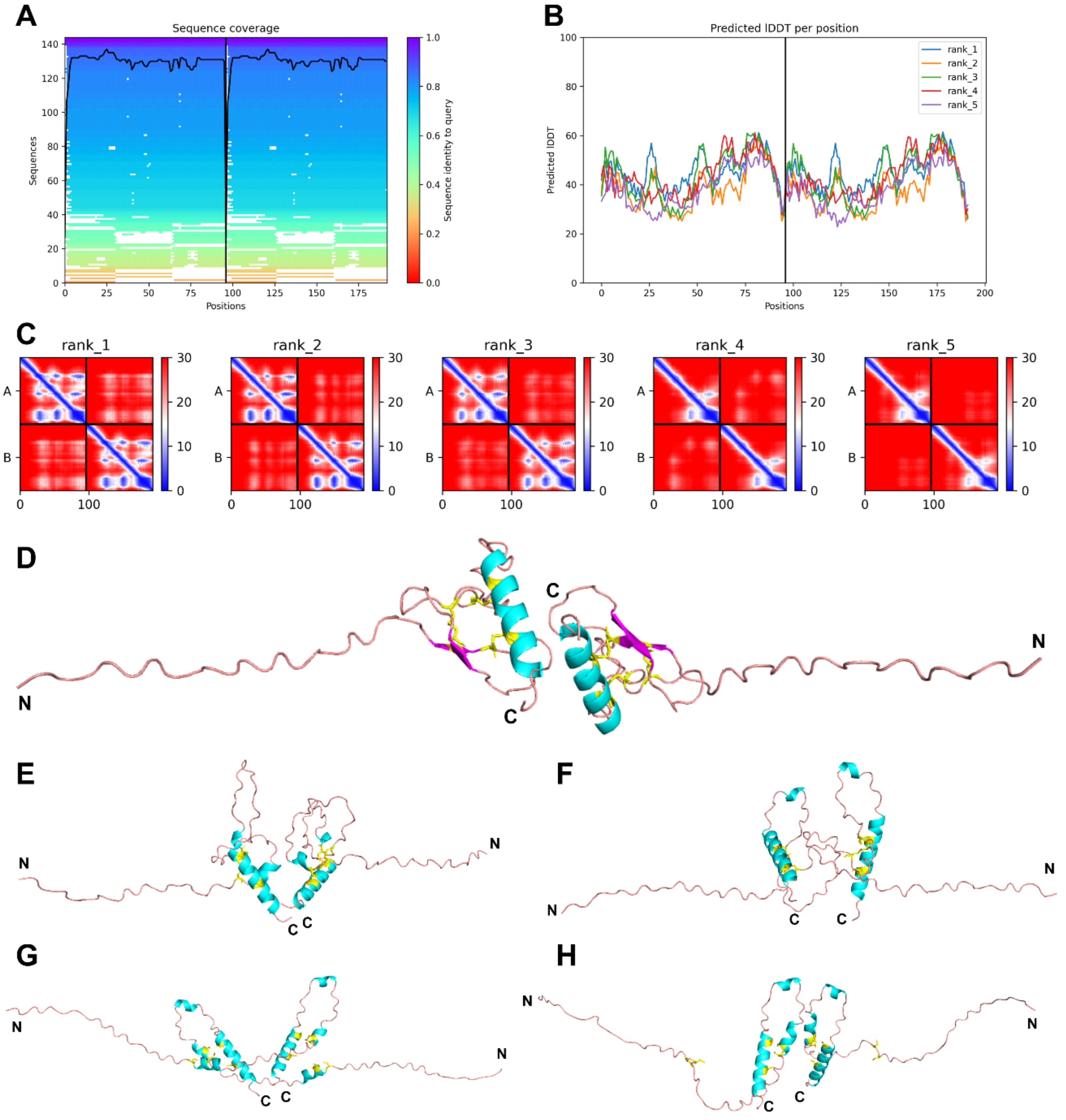

Supplementary Figure 5.

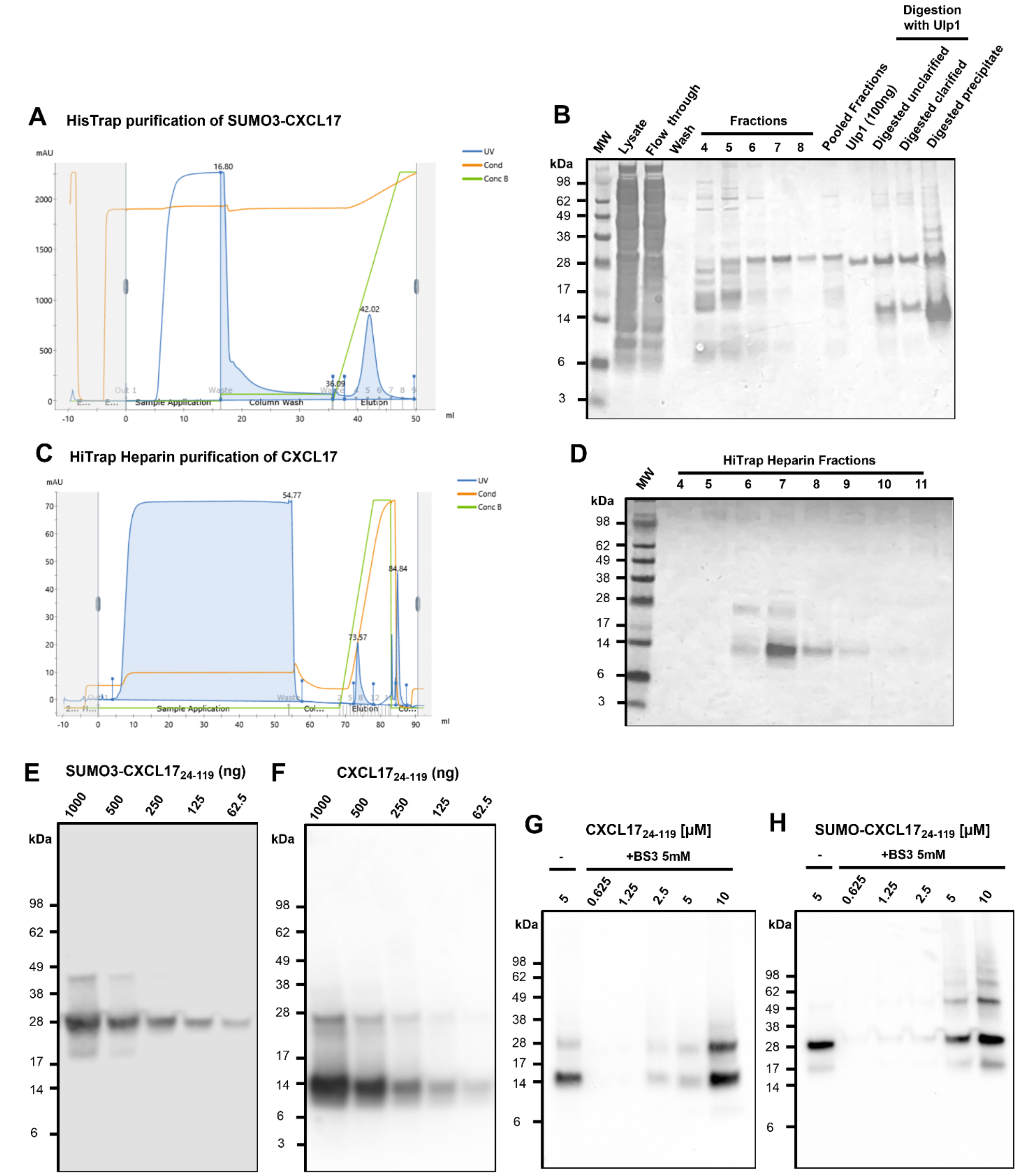

Supplementary Figure 6.

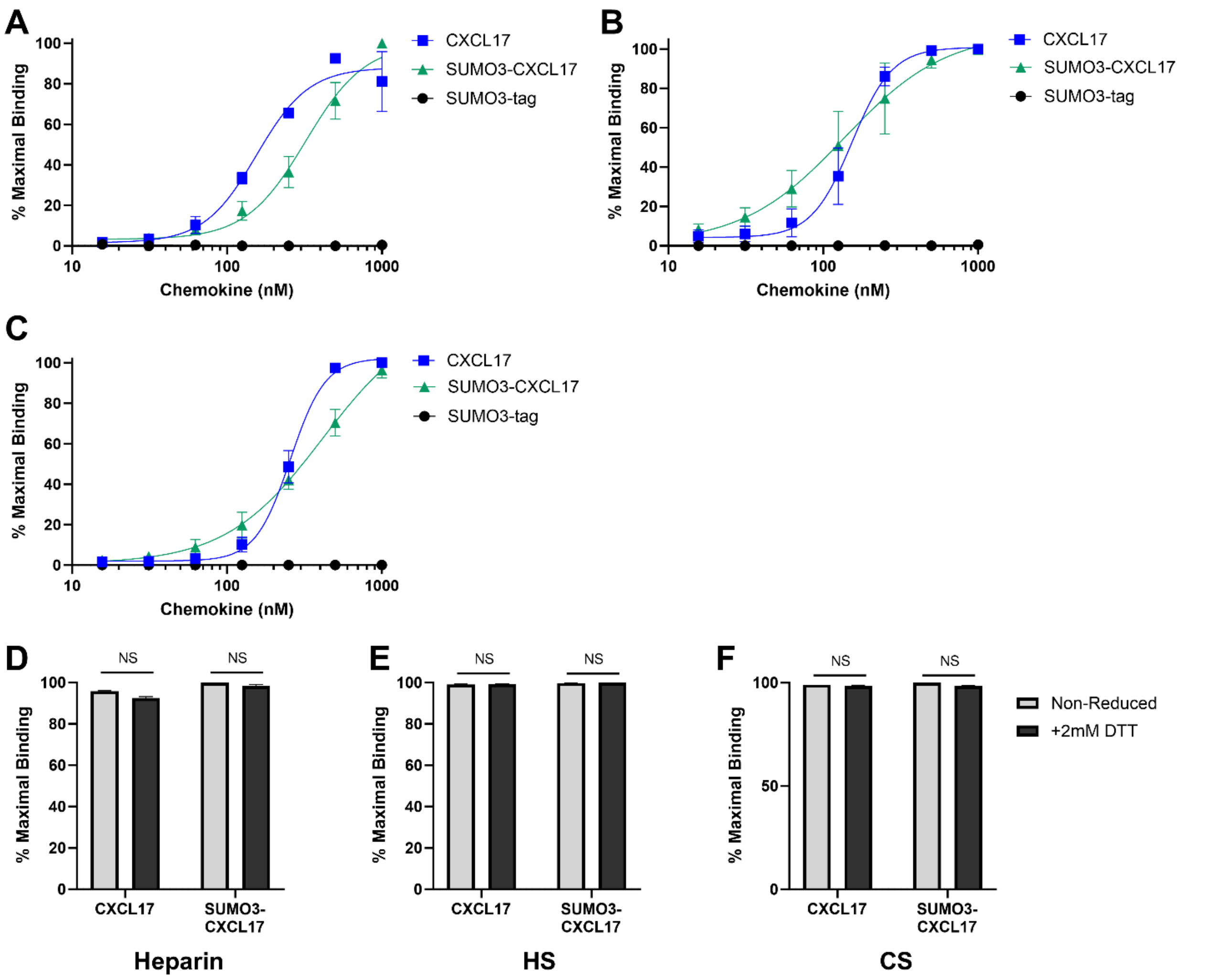

Supplementary Figure 7.
